# Chinese liquor extract enhances inflammation resistance in RAW 264.7 and reduces aging in *Caenorhabditis elegans*

**DOI:** 10.1039/c8ra06575f

**Published:** 2018-11-15

**Authors:** Jie Liu, Huailing Wang, Xiaoyu Liu, Guohao Zhang, Pingchang Yang, Zhigang Liu

**Affiliations:** Department of Allergy, The Third Affiliated Hospital of Shenzhen University Shenzhen 518020 China lzgszuniversity@sina.com +86-0755-86671907 +86-0755-86671907; The Research Center of Allergy & Immunology, Shenzhen University School of Medicine Shenzhen 518060 China

## Abstract

Recent reports have indicated that the ingredients in Chinese liquor possess multiple bioactivities. The objective of this study was to evaluate the potential effects of Chinese liquor extract (CME) on the resistance to inflammation in mononuclear macrophages (RAW 264.7 cell line) and aging in *Caenorhabditis elegans* (*C. elegans*). The results showed that CME suppressed key lipopolysaccharide (LPS)-induced pro-inflammatory mediators, tumor necrosis factor-α, and nitric oxide *in vitro*. Furthermore, CME inhibited activation of mitogen-activated protein kinase (MAPK) and phosphatidylinositol-3 kinase (PI3K)/AKT pathways in LPS-stimulated cells. Further studies also showed that CME improved stress resistance of nematodes under infection conditions. Moreover, CME increased the expression of immune-related genes, such as *lys-7*. Based on these results, our findings provide mechanistic insights about the protection provided by CME against LPS-induced inflammation in RAW 264.7 cells, namely, inhibition of MAPK and PI3K/AKT pathways, as well as its capability against *Pseudomonas aeruginosa*- and *Staphylococcus aureus*-induced aging in *C. elegans*.

## Abstract 2

Recent reports have indicated that the ingredients in Chinese liquor possess multiple bioactivities.

## Graphical abstract

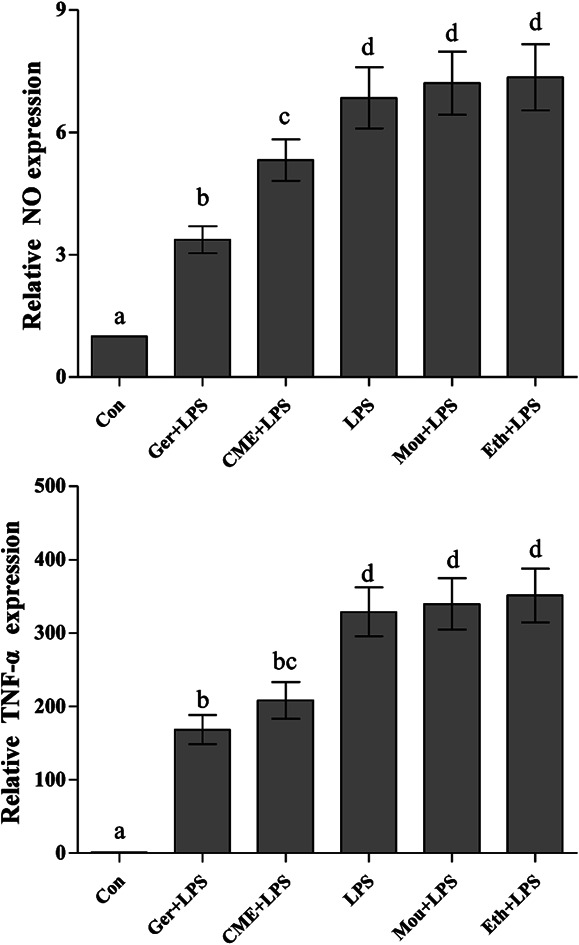

## Introduction

Components of the innate immunity include neutrophils and macrophages, and their role is to initiate an inflammatory response, form phagocytes to kill pathogens, recruit natural killer cells, and facilitate the maturation and migration of dendritic cells that will initiate the adaptive immune response.^1^ Inflammation, which is accompanied by fatal damages, such as oxidative damage, may worsen numerous diseases. However, inflammation is not typically a negative phenomenon: it is the response of the immune system to the invasion of viruses or bacteria and other pathogens. Emerging evidence suggests that inflammations are related to immunosenescence and reciprocally, controlling the inflammatory status may allow a better chance of controlling immunosenescence.^2^ Furthermore, inflammation is an important and necessary part of the normal host responses to pathogens, but the overproduction of inflammatory molecules might also cause immune-related inflammatory diseases and eventually death. Therefore, low responders involved in regulation of innate defense mechanisms might better control inflammatory responses and immunosenescence.^3^

Lipopolysaccharide (LPS), a pathogenic endotoxin that can induce inflammatory responses and oxidative stress, is widely used for evaluating anti-inflammatory efficacy. In particular, macrophages produce excess pro-inflammatory cytokines, tumor necrosis factor-α (TNF-α), and nitric oxide (NO) when exposed to inflammatory stimuli, such as LPS.^1^ Accumulating research shows that LPS causes overproduction of pro-inflammatory mediators and cytokines by activating nuclear factor-κB (NF-κB), which is associated with mitogen-activated protein kinase (MAPK) and phosphatidylinositol-3 kinase (PI3K)/AKT pathways.^2^ In addition, inflammatory stress increases the production of reactive oxygen species (ROS) and reduces antioxidant enzymes.^3,4^

*Caenorhabditis elegans* (*C. elegans*) is a commonly used model for aging as it exhibits the properties of a short lifespan, simple anatomy, and easy feeding.^5^ In addition, *C. elegans* share many highly conserved biochemical pathways with humans.^6^*Pseudomonas aeruginosa* (*P. aeruginosa*) and *Staphylococcus aureus* (*S. aureus*), two common pathogens in humans, are capable of causing a diverse range of diseases from superficial skin infections and soft tissue abscesses to life-threatening infections such as sepsis, endocarditis, pneumonia, and toxic shock syndrome.^7^ Moreover, bacterial infections shorten the lifespan of nematodes and cause immunosenescence.^8^

Aging is a process accompanied by deterioration of tissues, organs, and organism as well as the debasement of immunity, adaptability, and anti-infection ability, and finally the termination of life.^9^ There is a reciprocal relationship between aging and immune function degradation. Therefore, anti-aging treatments can improve immune function, while improving immune function can delay aging.^10^ Previous reports have shown that intake of antioxidants, such as natural products and herbal formulations, could enhance anti-infection ability and extend the lifespan of *C. elegans*.^11^

Chinese liquor is an alcoholic beverage obtained from grains by complex fermentation processes. Research studied the aroma compounds in Moutai liquor with comprehensive two-dimensional gas chromatography/time-of-flight mass spectrometry, and the results showed that a total of 528 components were identified in a Moutai liquor sample, including organic acids, alcohols, esters, ketones, aldehydes, acetals, lactones, and nitrogen-containing and sulfur-containing compounds, in addition to alcohol and water.^4^ Some studies have shown that Moutai liquor does not cause liver injury, but rather strengthen lipid peroxidation in the liver, induce the increase in metallothioneins (MT), inhibit the proliferation of hepatic stellate cell (HSC) and generation of collagen and enhance the effect of antioxidation owing to multiple bioactive substances present in it.^5^ Chinese liquor has historically been regarded as the most important Chinese medicine by herbalist doctors.^12^

Recent reports have indicated that some substances in Chinese liquor, such as geraniol, possess a variety of bioactivities such as anticancer, anti-inflammatory, and antibacterial properties.^13^ Chinese liquor has been consumed in China for thousands of years, and numerous studies have reported that some ingredients in Chinese liquor have antiseptic, anti-inflammatory, and antioxidant effects; however, no previous study has focused on the anti-infection effect of Chinese liquor. As infection-related morbidity and mortality is closely associated with environmental stress in a given population, potential stress-resistance effects of CME in *C. elegans* were also investigated in this paper. Evaluating the protective effect of CME and its underlying mechanisms will broaden the understanding of Chinese liquor and provide a reference for healthy drinking.

## Materials and methods

### Chemicals and reagents

Chinese liquor (53% v/v) was obtained from KweiChow Moutai Distillery in Guizhou province, China. Geraniol (Ger), lipopolysaccharide (LPS), 2′,7′-dichlorofluorescin diacetate (DCFH-DA), 5-fluoro-20-deoxyuridine (FUDR), and Paraquat were purchased from Sigma-Aldrich (St. Louis, MO, USA). All antibodies were purchased from Cell Signaling Technology (Danvers, MA, USA). The RAW 264.7 cells were obtained from the Cell Bank of the Chinese Academy of Sciences (Shanghai, China).

### Chinese liquor extract preparation

Chinese liquor extract (CME) samples were prepared by the following method. Initially, 50 mL Chinese liquor, 20 mL H_2_O, and 90 mL CHCl_3_ were added to a 250 mL separating funnel. The CHCl_3_ layer was evaporated at 0 °C using a Turbovap Sample Concentrator. Approximately 0.39 mg of the residue remained and was taken as CME and stored at −20 °C in the dark. Mentions of brand names do not imply any research contact with the liquor manufacturer nor are these mentions for advertising purposes.

### Cell and nematode culture

RAW 264.7 cells were maintained in Dulbecco's Modified Eagle's Medium containing 10% (v/v) fetal bovine serum, l-glutamine (2 mM), penicillin (100 U mL^−1^) and streptomycin (100 U mL^−1^) at 37 °C in a humidified atmosphere containing 5% CO_2_; culture medium was changed every 2 days. Cells in logarithmic growth phase were selected for experiments and divided into the following six experimental treatment groups: control, LPS, ethanol + LPS, Mou + LPS, CME + LPS, and Ger + LPS. Nematodes were cultured at 25 °C on nematode growth medium (NGM) plates and seeded with *Escherichia coli* OP50. Lyosgeny broth and slow-killing medium were prepared according to previously reported methods.^14^

### Determination of intracellular ROS and NO in RAW 264.7 cells

The cytotoxic activity of CME was detected using a previously described method,^15^ while intracellular ROS production was detected with a DCFH-DA using another previously reported method.^16^ Briefly, RAW 264.7 cells were pre-treated with chemicals for 24 h, and then stimulated with LPS (100 μg mL^−1^). After incubation for 24 h, the cells were stained with 10 μM DCFH-DA at 37 °C in the dark. After 0.5 h, the cells were washed with phosphate-buffered saline (PBS) twice, and then placed in an Ascent FL fluorescence plate reader (37 °C). Emission at 535 nm was measured after excitation at 485 nm for 20 min. For NO concentration detection, cells were cultured using the same method before collecting cell supernatants. NO concentration was detected using a commercial NO ELISA kit (Beyotime, Shanghai, China) according to the manufacturer's instructions.

### Western blot analysis

RAW 264.7 cells were incubated with chemicals [geraniol, CME, Chinese liquor (53%), ethanol (v : v/ethanol : H_2_O/53 : 47)] for 24 h prior, and then stimulated with LPS (100 μg mL^−1^) for 24 h. After incubation, the cells were collected, washed with PBS, lysed in 100 mL lysis buffer containing protease and phosphatase inhibitor cocktails, and centrifuged at 13 000×*g* for 15 min. An immunoblotting assay was performed as previously reported using β-actin protein as a measurement of the amount of protein analyzed.^17^

### Nematode lifespan assays

Nematodes were maintained on NGM plates and seeded with *Escherichia coli* OP50 at 25 °C to obtain L4-larvae nematodes, as previously reported.^18^ Young adult worms were transferred to NGM plates containing FUDR (50 μM) to prevent the growth of progeny. This transfer day was designated as day 0. For the safety assessment, CME, geraniol, Chinese liquor and ethanol were added to NGM plates (100 μL per plate) at concentrations ranging from 0–0.40 mg mL^−1^ for 48 h before detecting effects on lethality, growth, and movement behavior using previously reported methods.^19^

### Anti-*Pseudomonas aeruginosa* and anti-*Staphylococcus aureus* infection assays

Experiments were performed according to a previously reported method with some modifications.^20^ The minimal inhibitory concentration (MIC) of CME, geraniol, Chinese liquor and ethanol against *P. aeruginosa* and *S. aureus* was detected using a previously reported method.^21^ Briefly, young adult worms were transferred to new NGM plates containing FUDR (50 μM) to prevent the growth of progeny, and then pre-treated for 48 h in the following groups: blank (Con); ethanol (V : V/ethanol : H_2_O/53 : 47) (Eth); Chinese liquor (53%) (Mou); CME (CME); and geraniol (Ger). Synchronized young adult nematodes were cultured in NGM plates with or without chemicals for 24, 48, or 72 h. After incubation, approximately 80 nematodes were randomly picked and placed into plates coated with *P. aeruginosa* or *S. aureus*, which were cultured at 25 °C; this day was considered as day 0. Each day, the number of surviving nematodes was counted until all nematodes were dead. Triplicate plates were used for each group. Nematodes that showed no movement on touching gently were considered dead.

### Real time-polymerase chain reaction (RT-PCR) assays

Nematodes were treated as described above. After pre-treatment with CME for 48 h and infection with *P. aeruginosa* or *S. aureus* for 48 h, total RNA was isolated from about 1500 nematodes using Trizol reagent.^22^ Primer sequences for RT-PCR are listed in Table 1 and results are reported as mean ± SD.

**Table 1:** Primer sequence of RT-PCR

Gene	Primer sequence
*lys-7*	F: 5′-GTCAAGGTTCCCCCGATTGT-3′
	R: 5′-ATCCTTGTCCTGCTGGGTTG-3′
*clec-60*	F: 5′-ATAGGTTGTGGCGTATGGGC-3′
	R: 5′-AATGTTCAATCGGCCACCCT-3′
*f08g5.6*	F: 5′-TGTCCCACTGTCACAAGCTC-3′
	R: 5′-TCGGGAATTGGGTTTCGACC-3′

## Results and discussion

### Cytotoxicity of CME on RAW 264.7 cells

After obtaining the CME, we analyzed it with GC-MS, and the results showed that the CME is mainly composed of esters, polyols, acids, aromatic compounds, furans, aldehydes and pyrazines. HPLC analysis showed that the concentration of geraniol in Chinese liquor is 1.107 ± 0.013 μg L^−1^ (*p* > 0.05).

In consideration of the fact that compounds may themselves elicit cytotoxicity, the effect of each chemical on RAW 264.7 cell viability was tested. The results showed that no chemical tested (maximum concentration 50.0 μg mL^−1^) had a significant effect on RAW 264.7 cell viability. Accordingly, the anti-inflammatory effects of CME on RAW 264.7 cells were detected at 50.0 μg mL^−1^.

### Effects of CME on pro-inflammatory mediators

**Fig. 1:**
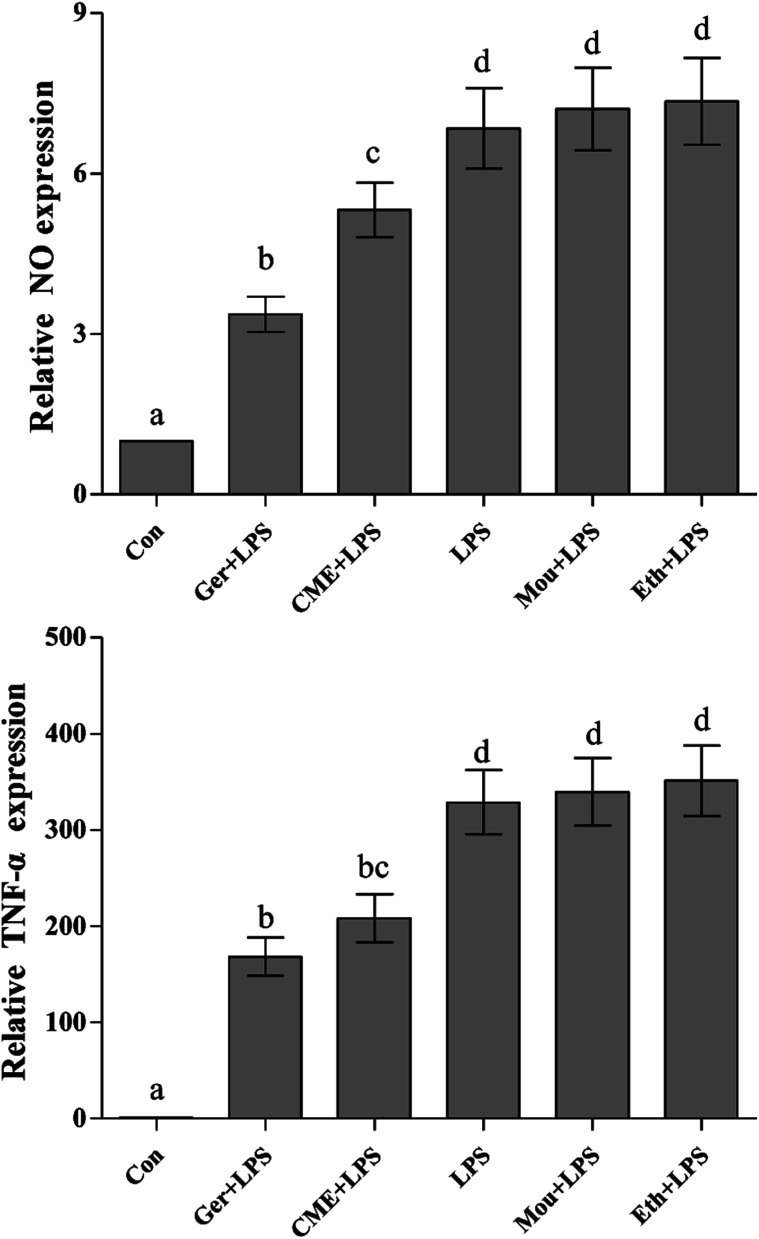
CME represses LPS-induced NO and TNF-α production. RAW264.7 cells were pretreated with the indicated reagents for 24 h, and then stimulated with LPS for 24 h. NO and TNF-α levels were measured as described in the Materials and methods. (Con): DMEM only; (Ger + LPS): geraniol at 50.0 μg mL^−1^ for 24 h and then with LPS at 100.0 μg mL^−1^ for 24 h; (CME + LPS): CME at 50.0 μg mL^−1^ for 24 h and then with LPS at 100.0 μg mL^−1^ for 24 h; (LPS): LPS at 100.0 μg mL^−1^ for 24 h; (Mou + LPS): Chinese liquor (53%) at 50.0 μg mL^−1^ for 24 h and then with LPS at 100.0 μg mL^−1^ for 24 h; (Eth + LPS): ethanol (V : V/ethanol : H_2_O/53 : 47) at 50.0 μg mL^−1^ for 24 h and then with LPS at 100.0 μg mL^−1^ for 24 h. Data are represented as the mean ± SD (*n* = 3). Bars with different letters indicate significant difference between groups (*p* < 0.05).

NO, a representative pro-inflammatory mediator, is regulated by inducible NO synthase (iNOS).^23^ In addition, major pro-inflammatory cytokines, such as TNF-α, are overexpressed in macrophages stimulated by LPS, where they contribute to the pathogenesis of various inflammatory diseases.^24^ After treatment with LPS, uncontrolled activation of RAW 264.7 cells can cause injury *via* overproduction of the inflammatory mediators such as NO and TNF-α,^25^ which is consistent with the results presented in Fig. 1 (NO and TNF-α content in the control group was set as 1). These results indicated that LPS stimulation increased the NO concentration (6.85 ± 0.75) compared with the control group, but was significantly reduced by CME (5.32 ± 0.51). In addition, TNF-α was significantly reduced on CME pre-treatment (208.3 ± 25.2) compared with that observed in the LPS group (328.9 ± 33.4). These results suggested that CME played a significant role in modulating inflammatory cytokine expression. Geraniol showed a similar effect to CME: it reduced the concentration of NO by 50.8% and inhibited TNF-α protein expression by 48.7% compared with that of the LPS-administered group. Compared with the LPS group, the relative NO concentrations and TNF-α protein expressions in Chinese liquor and ethanol groups increased, but showed no significant difference.

### CME reduced ROS levels

**Fig. 2:**
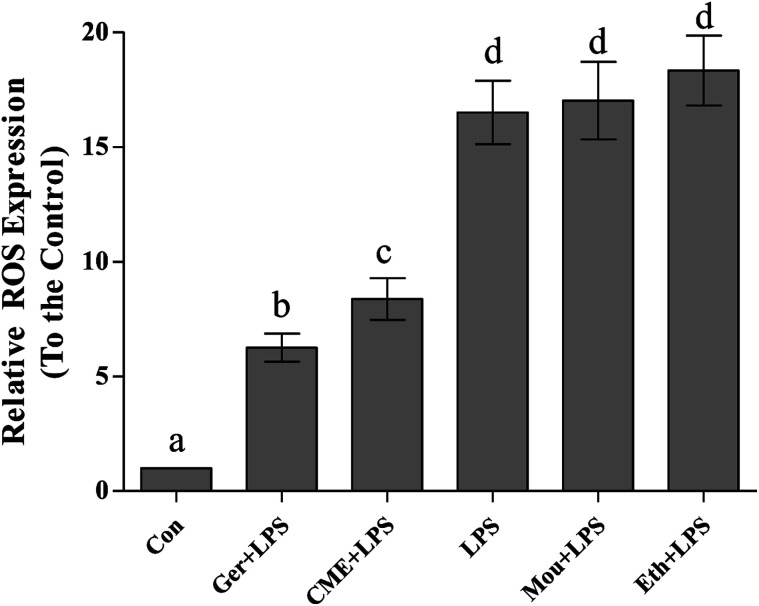
CME represses LPS-induced ROS production. RAW264.7 cells were pretreated with the indicated reagents for 24 h, and then stimulated with LPS for 24 h. ROS production was measured as described in the Materials and methods. (Con): DMEM only; (Ger + LPS): geraniol at 50.0 μg mL^−1^ for 24 h and then with LPS at 100.0 μg mL^−1^ for 24 h; (CME + LPS): CME at 50.0 μg mL^−1^ for 24 h and then with LPS at 100.0 μg mL^−1^ for 24 h; (LPS): LPS at 100.0 μg mL^−1^ for 24 h; (Mou + LPS): Chinese liquor (53%) at 50.0 μg mL^−1^ for 24 h and then with LPS at 100.0 μg mL^−1^ for 24 h; (Eth + LPS): ethanol (V : V/ethanol : H_2_O/53 : 47) at 50.0 μg mL^−1^ for 24 h and then with LPS at 100.0 μg mL^−1^ for 24 h. Data are represented as the mean ± SD (*n* = 3). Bars with different letters indicate significant difference between groups (*p* < 0.05).

Inflammation and oxidative stress are now recognized as two important factors contributing to the development of many diseases, and ROS plays crucial roles in both processes.^26^ Indeed, oxidative stressors would cause serious damage to lipids, proteins, and DNA.^27,28^ Intake of red wine can naturally interrupt ROS expression and inhibit the progression of inflammation and oxidative stress.^29^ As shown in Fig. 2, relative ROS expression in the LPS group (16.5 ± 1.38) significantly increased compared with that in the control group. Pre-treatment with CME or geraniol significantly reduced LPS-induced ROS expression (8.37 ± 0.91 and 6.25 ± 0.62, respectively). In contrast to CME and geraniol, pre-treatment with ethanol or Chinese liquor increased relative ROS expression to 18.3 ± 1.52 and 17.0 ± 1.69, respectively. As the results showed that CME and geraniol reduced ROS expression, we inferred this effect may be associated with its antioxidant activity. However, neither ethanol nor Chinese liquor showed protective effects and instead increased the level of ROS. We inferred that the main component, ethanol, elicited these harmful effects and induced ROS expression.

### CME inhibits NF-κB and depresses IκB-α pathways

**Fig. 3:**
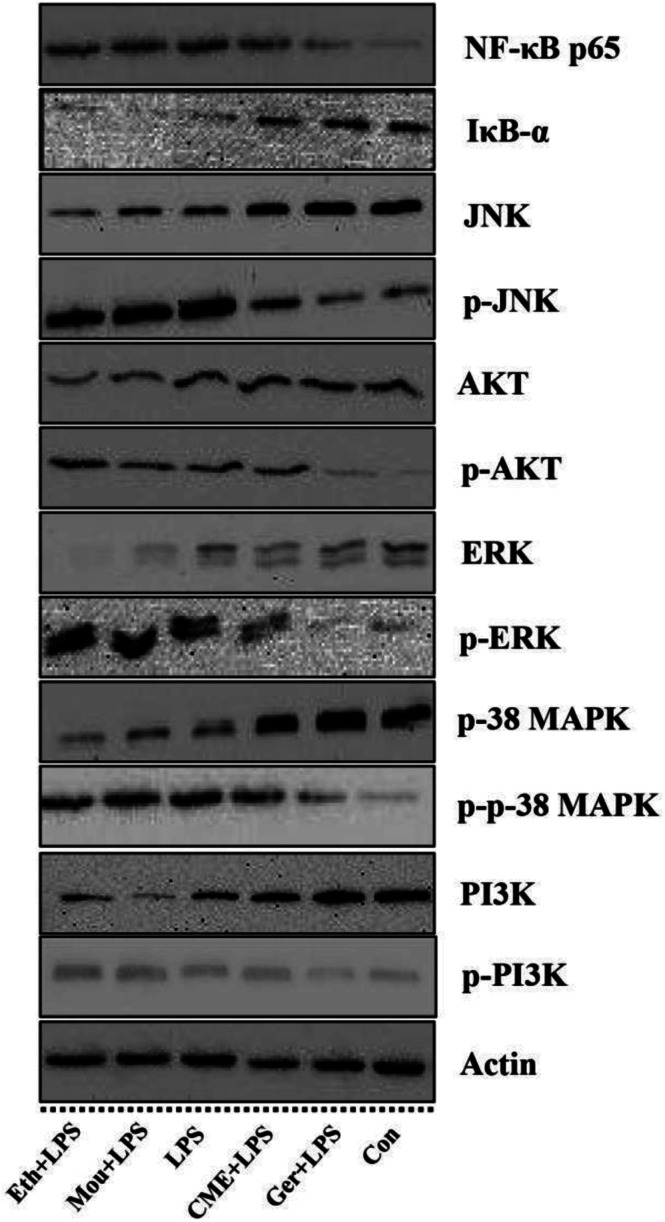
The relative expression level of proteins including: NF-κB p65, IκB-α, JNK, p38 MAPK, ERK, PI3K, AKT, p-JNK, p-p38 MAPK, p-ERK, p-PI3K, p-AKT, and proteins in mononuclear macrophage RAW264.7 cells. Protein assay results were confirmed by western blotting, and the relative expression levels of the proteins were determined. (Con): DMEM only; (Ger + LPS): geraniol at 50.0 μg mL^−1^ for 24 h and then with LPS at 100.0 μg mL^−1^ for 24 h; (CME + LPS): CME at 50.0 μg mL^−1^ for 24 h and then with LPS at 100.0 μg mL^−1^ for 24 h; (LPS): LPS at 100.0 μg mL^−1^ for 24 h; (Mou + LPS): Chinese liquor (53%) at 50.0 μg mL^−1^ for 24 h and then with LPS at 100.0 μg mL^−1^ for 24 h; (Eth + LPS): ethanol (V : V/ethanol : H_2_O/53 : 47) at 50.0 μg mL^−1^ for 24 h and then with LPS at 100.0 μg mL^−1^ for 24 h. Bars with different letters mean significant difference between groups (*p* < 0.05).

**Fig. 4:**
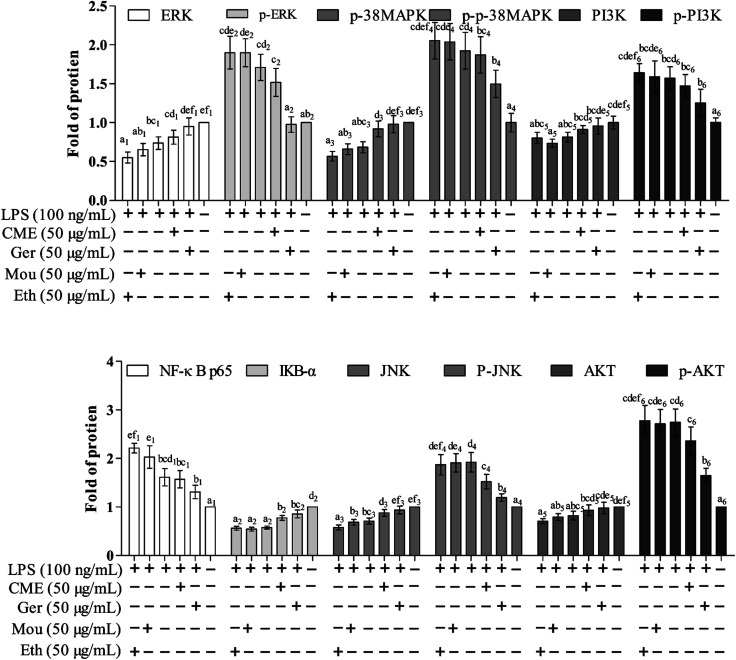
Effect of CME on proteins related to NF-κB, MAPKs and PI3K/AKT pathways in LPS-induced RAW264.7 cells. Actin was used as an internal control. RAW264.7 cells were treated with indicated reagents. (Con): DMEM only; (Ger + LPS): geraniol at 50.0 μg mL^−1^ for 24 h and then with LPS at 100.0 μg mL^−1^ for 24 h; (CME + LPS): CME at 50.0 μg mL^−1^ for 24 h and then with LPS at 100.0 μg mL^−1^ for 24 h; (LPS): LPS at 100.0 μg mL^−1^ for 24 h; (Mou + LPS): Chinese liquor (53%) at 50.0 μg mL^−1^ for 24 h and then with LPS at 100.0 μg mL^−1^ for 24 h; (Eth + LPS): ethanol (V : V/ethanol : H_2_O/53 : 47) at 50.0 μg mL^−1^ for 24 h and then with LPS at 100.0 μg mL^−1^ for 24 h. The proteins expression levels were measured using western blot. The density of each lane was presented as mean ± SD for at least three individual experiments. Blots were quantified using Image J software. Bars with different letters indicate significant difference between groups (*p* < 0.05).

The NF-κB signaling pathway is important for inflammatory responses. Activation of NF-κB requires phosphorylation of upstream IκB kinase (IKK), which contains two catalytic subunits, IKKα and IKKβ.^30^ Upon stimulation with LPS or pro-inflammatory cytokines, IKK is phosphorylated and activated by TGF-β-activated kinase 1 (TAK1), resulting in further phosphorylation and depression of IκB in the ubiquitination pathway. Subsequently, NF-κB releases from the IκB/NF-κB dimer and translocates from the cytoplasm into the nucleus, inducing further pro-inflammatory gene expression and inflammatory responses. Therefore, inhibition of the NF-κB pathway may have a potential therapeutic effect. As such, the effect of CME on NF-κB pathway inhibition was investigated. As shown in Fig. 3 and 4, LPS stimulation markedly increased NF-κB p65 expression by 61.0% and reduced IκB-α expression by 42.7% compared with those in the control group. However, with CME pre-treatment, NF-κB p65 expression was markedly inhibited and IκB-α expression was upregulated. Compared with the LPS group, expression of NF-κB p65 was reduced by 11.8% and that of IκB-α was increased by 35.1%. These results suggest that CME may act as a negative regulator of NF-κB activation. However, ethanol and Chinese liquor showed contrasting effects as they increased NF-κB p65 expression and reduced IκB-α expression compared with those in the LPS group. We inferred that the ethanol in these groups played the main functions.

### CME inhibits activation of MAPK and PI3K/AKT pathways

MAPKs play significant roles against inflammation, and extracellular signal-regulated protein kinase (ERK) can regulate, at least in part, NO production, iNOS expression, and TNF-α secretion.^31^ Moreover, rapid and lasting activation of c-Jun NH(2)-terminal kinase MAPK (JNK) can be induced by LPS in RAW 264.7 cells, and inhibition of JNK reduces LPS-induced TNF-α activity. A previous study has also shown that the PI3K/AKT signaling pathway is involved in inflammation.^32^ As shown in Fig. 3 and 4, LPS stimulation promoted the phosphorylation of JNK, p38 MAPK, ERK, as well as PI3K and AKT by approximately 92.5%, 92.1%, 71.3%, 57.1%, and 174%, respectively, compared with those in the control group. Moreover, LPS inhibited the expression of JNK, p38 MAPK, ERK, PI3K, and AKT. Furthermore, with CME treatment, JNK, p38 MAPK, ERK, PI3K, and AKT expression increased, and their phosphorylation was inhibited compared with LPS groups. Thus, we concluded that CME may suppress the inflammatory response by inhibiting MAPK and PI3K/AKT pathways. However, both ethanol and Chinese liquor showed contrasting effects to CME, and played pro-inflammatory roles in response to LPS.

### CME enhanced the anti-infection ability of nematodes

*C. elegans* is a popular animal model for aging studies because it has a short lifespan, rapid generation, and experimental flexibility. In addition, *C. elegans* shares highly conserved biochemical pathways and many similar aging aspects with humans. From the initial identification of single gene mutations capable of increasing aging to the insulin-like growth factor signaling pathway, molecular mechanisms of aging have gradually become an increased focus of research. *C. elegans* has become the major model organism contributing to the discovery of molecular mechanisms of aging.

As previously reported, antioxidants increase the lifespan of nematodes, and some non-ethanol ingredients in Chinese liquor, such as geraniol, exhibit good antioxidant activity *in vitro*.^33^ Resveratrol, a polyphenol antioxidant found in red wine, has been the subject of intense interest in recent years for a range of unique anti-aging properties.^34^ Blueberry wine with proanthocyanidin also exhibited optimal anti-aging effects on *C. elegans*.^35^ Clinically, alcohol is used for sterilization. In general, the substances in Chinese liquor may inhibit bacterial growth, thus affecting the lifespan of nematodes. Therefore, we tested the MIC of CME, geraniol, Chinese liquor and ethanol, to avoid this hypothesis. We found that treatment with 0–0.40 mg mL^−1^ of CME, geraniol, Chinese liquor and ethanol did not affect the growth of bacteria or development, brood size, movement behavior, or viability of nematodes. Thus, a concentration of 0.20 mg mL^−1^ CME, geraniol, Chinese liquor and ethanol were selected for subsequent studies.

**Fig. 5:**
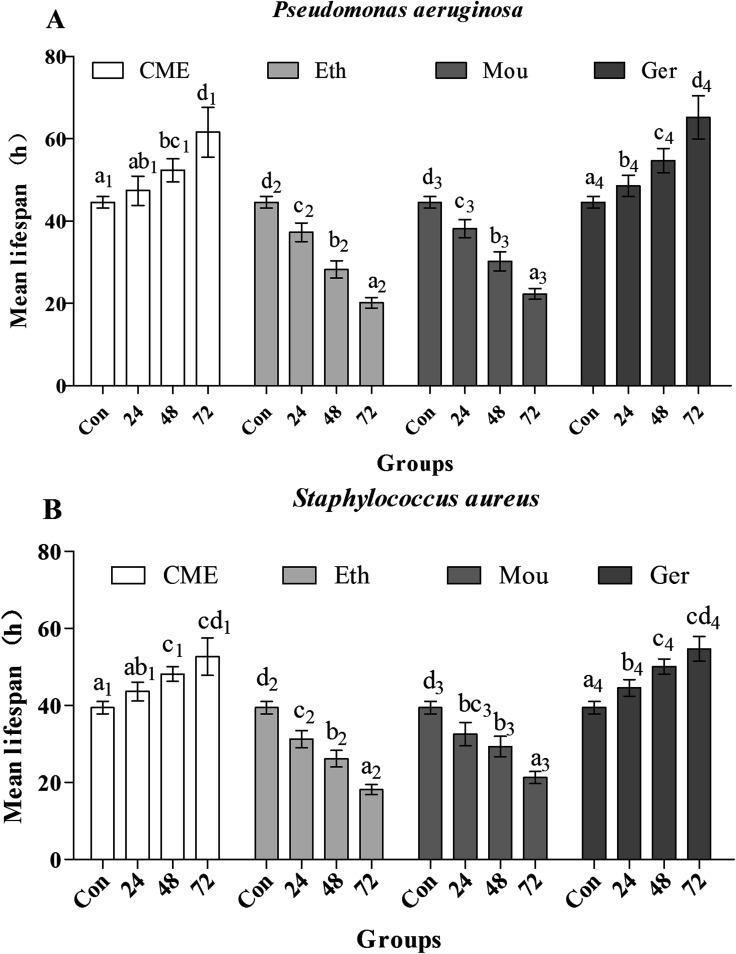
Effect of CME treatment on anti-aging in *C. elegans*. The young adult nematodes were treated with CME for 24, 48 and 72 h, then exposed to *Pseudomonas aeruginosa* (A) and *Staphylococcus aureus* (B) and monitored the survivals every hour (*N* ≥ 80 animals). The experiment was repeated multiple times and a representative trial is shown. Bars with different letters indicate significant difference between groups (*p* < 0.05).

After pre-treatment with CME for 24, 48, or 72 h, lifespans were greatly improved, as shown in Fig. 5. Mean lifespans in control groups were 44.6 ± 1.40 h and 39.5 ± 1.60 h after infection with *P. aeruginosa* or *S. aureus*, respectively. However, with CME pre-treatment, mean lifespans in infected groups increased. With CME pre-treatment for 24, 48, or 72 h, mean lifespans were 47.4 ± 3.60 h, 52.3 ± 2.80 h, and 61.6 ± 6.00 h in *P. aeruginosa*-infected groups, respectively; compared with the control group, the mean lifespans increased by 6.37%, 17.4%, and 38.2%, respectively. In addition, the mean lifespans of nematodes in the *S. aureus*-infected groups were also enhanced to 43.6 ± 2.50 h, 48.2 ± 1.90 h, and 52.7 ± 4.90 h with CME pre-treatment for 24, 48, and 72 h respectively; compared with the control group, the lifespans increased by 10.5%, 22.0%, and 33.5%, respectively. For the ethanol and Chinese liquor groups, the lifespans of nematodes did not extend, but instead shortened. We inferred that the presence of ethanol may play a harmful effect and weakened the anti-stress ability of nematodes.

### Genetic requirements for increased anti-infection with CME treatment

**Fig. 6:**
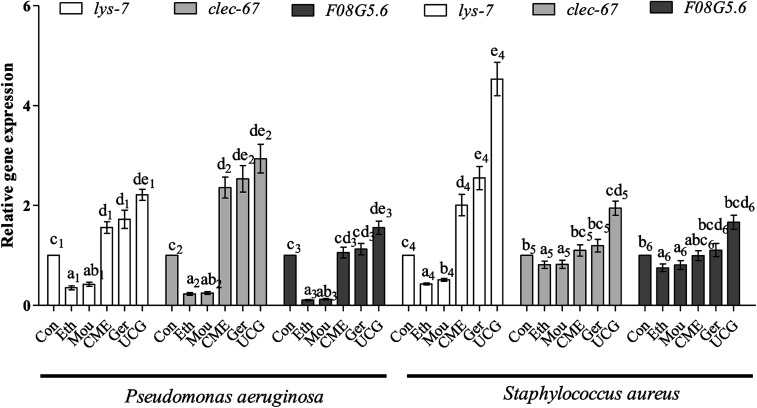
The relative expression level of mRNA including: *lys-7*, *clec-60*, *F08G5.6* in *C. elegans*. Assay results were confirmed by RT-PCR, and the relative expression levels of the mRNA were determined. Bars with different letters indicate significant difference between groups (*p* < 0.05).

Bacteria are capable of causing disease and infection, which can abbreviate the lifespan of nematodes and cause aging. Expression levels of immune and aging effector genes significantly changed upon bacterial infection. During the initial stage of infection, C-Type lectin genes (*e.g.*, *clec-60*) appear to be responsible for enhanced adhesion and antibacterial activity.^36^ The lysozyme gene *lys-7*, a presumptive antimicrobial gene critical for the induction of immune response, is upregulated in response to bacterial infection. Regulation of *lys-7* mRNA against bacterial infection gradually increases with time of exposure and upregulation.^37^ The *F08G5.6* gene belongs to a family of CUB-like proteins. Previous reports support the role for proteins carrying CUB-like domains in immunity against pathogens in *C. elegans*. The *F08G5.6* gene is regulated by daf-2/daf-16 and p38 MAPK pathways.^38^ After pre-treatment with CME for 48 h, nematodes were infected with *P. aeruginosa* or *S. aureus* for 48 h. Expression levels of immune-related genes were then tested by RT-PCR. The results are shown in Fig. 6. For the *P. aeruginosa* infection condition, relative expression of *lys-7* and *clec-60* were upregulated 1.56- and 2.36-fold, respectively, in the CME treatment group compared with that in the control group. However, *F08G5.6* expression was not different from the CME treatment group (1.05-fold).

For *S. aureus* infection conditions, relative expression of *lys-7* was upregulated (2.00-fold) in the CME treatment group compared with that in the control group. However, expression of *F08G5.6* and *clec-60* were not different in the CME treatment groups (1.10- and 0.99-fold). Notably, geraniol showed better protective effect than CME, but both ethanol and Chinese liquor showed contrary effects, where the relative expression of immune-related genes *lys-7*, *clec-60*, and *F08G5.6* was downregulated compared with that of the control group. Expression of immune genes is closely related to the anti-infection ability of *C. elegans*. The present study showed that CME upregulated the relative expression of *lys-7* during *P. aeruginosa* and *S. aureus* infections. Thus, we inferred that the immune gene *lys-7* played a role in stress resistance against bacterial infection. However, according to the results listed in Fig. 6, the relative expressions of *lys-7*, *clec-60*, and *F08G5.6* in the uninfected control groups (UCG) were higher than those in the CME and geraniol groups. We inferred that treatments can attenuate but not offset the harm of bacteria.

## Conclusions

Chinese liquor, one of the famous distilled liquors, is consumed worldwide and has components with diverse bioactivities. CME suppressed LPS-induced production of the key pro-inflammatory mediators NO and TNF-α *in vitro*. In addition, the MAPK/PI3K/AKT signaling pathway was inhibited by CME treatment in LPS-induced RAW 264.7 cells. Moreover, CME upregulated the expression of immune-related genes and enhanced the anti-infection ability of cells. Future investigations will aim to provide better understanding of the function of Chinese liquor. Indeed, both animal and human studies must be designed, as extrapolations cannot be made from cell culture studies to humans.

## Conflicts of interest

The authors declare no conflict of interest.

## Supplementary Material
